# Assessment of the outcomes and stability after mandibular incisor extraction in orthodontic patients: A systematic review and meta-analysis

**DOI:** 10.34172/joddd.2023.36989

**Published:** 2023-07-17

**Authors:** Rasiga Gandhi, Poornima Jnaneshwar, Keerthi Venkatesan, Davis Devasahayam, Krishnaraj Rajaram, Rajia Mohamed Azharudeen, Kavichithraa Jothy

**Affiliations:** ^1^Department of Orthodontics and Dentofacial Orthopaedics, SRM Dental College, Ramapuram, Chennai, Tamilnadu, India; ^2^Department of Orthodontics and Dentofacial Orthopaedics, Faculty of Dental sciences, SRIHER, Chennai, Tamilnadu, India; ^3^Orthodontist, Private Practice, Kadayanallur, Tenkasi, Tamilnadu, India; ^4^Department of Orthodontics, Adhiparasakthi Dental College & Hospital, Melmaruvathur, Chengalpattu, India

**Keywords:** Intercanine width, Mandibular incisor extraction, Orthodontic patients, PAR index, Relapse, Stability

## Abstract

**Background.:**

This study assessed the stability of the outcomes after mandibular incisor extraction (MIE) using intercanine width and peer assessment rating (PAR) scores in orthodontic patients.

**Methods.:**

PubMed, Cochrane Library, Science Direct, Google Scholar, Ovid, and SciELO were systematically searched without restrictions until August 2022. A risk of bias assessment was performed using Newcastle-Ottawa Scale (NOS). The Grading of Recommendations, Assessment, Development, and Evaluation tool was used to assess the quality of evidence. Random effects meta-analysis was performed using RevMan software.

**Results.:**

Seven retrospective studies met the inclusion criteria and were included. Meta-analysis identified a statistically significant reduction in intercanine width with MIE after the retention period. The mean difference in post-retention changes concerning intercanine width (MD=0.14, 95% CI: -2.17–1.89; *P*<0.00001) was significantly higher in premolar extraction (PE) compared to incisor extraction and significantly less in non-extraction compared to incisor extraction (MD=0.72, 95% CI: -0.59–2.03; *P*<0.00001). Improvements in PAR scores from the start of treatment to the retention period indicated a high outcome standard (>70%) with MIE treatment, with no significant difference in the reduction percentage compared to premolar and non-extraction groups.

**Conclusion.:**

With the existing retrospective studies of limited evidence, treatment outcomes with MIE were found to show good improvements in PAR scores. Some reduction in the intercanine width was evident after the retention period, which was observed even with the other two treatment modalities that were compared. Hence, with careful evaluation, MIE could be considered a valid treatment option.

## Introduction

 The debate of extraction versus non-extraction approach in orthodontic treatment has always been long-standing. The treatment trends were observed to fluctuate between non-extraction and extraction of four premolars.^1^ Mandibular incisor extraction (MIE) has been reported as a rare extraction choice in orthodontic treatment with a frequency ranging from 2.1%^2^ to 6%.^3^

 The concept was first proposed by Hahn,^4^ and extraction of single or even two incisors was presented in literature predominantly as case reports and case series. Kokich and Shapiro recommended that with careful planning, case selection, and a complete diagnostic setup, intentional extraction of lower incisors can lead to good results with minimal orthodontic manipulation.^5^ The following indications have been strongly recommended for MIE: class I molar relationship, moderate crowding in mandibular anterior teeth, mild or no crowding in the maxillary anterior teeth, acceptable soft tissue profile, minimal to moderate overjet and overbite, minimal growth potential and tooth size discrepancies (peg laterals, missing laterals).^6,7^ It has been suggested as a good treatment alternative in mild to moderate class III malocclusion with reduced overjet and overbite.^8^

 Many authors object to the treatment plan of incisor extraction, citing unwanted treatment effects, including non-coincidental midlines,^9^ increased overjet and overbite, mesial tipping of canine, which can cause difficulties in achieving class I canine relationship, lingually tipped incisors, inadequate space, excess space creation and space reopening,^10^ relapse of crowding of the incisors,^11^ unfavorable posterior occlusion,^12^ loss of interdental papillae, and appearance of less aesthetic black triangles.^13^ This led to the description of MIE as a compromised treatment approach. However, some authors considered it an ‘acceptable compromise’ and a valid treatment option though ideal occlusion standards were not met.^14^ MIE has been advocated in orthodontic practice with fixed appliances to date and has also been carried out with Invisalign treatment.^15^

 Although the treatment looks successful from the above-discussed perspective, actual treatment success lies in the stability of achieved results. The view on stability appears controversial as contradictory statements were found, with some reporting stable results,^7,16-18^ while others reported instability and promoted the need for long-term retention.^19^ The presence of lingually bonded retainers resulted in less relapse than in patients without a retainer.^20^

 Valinoti reported that lower incisor extraction is less likely to exhibit relapse after retention based on factors such as i) proximity of incisor to crowding, requiring minimal tooth movement, and preserving larger areas of the original position of teeth; ii) less load on anchor teeth during space closure utilizing most space for anterior correction; iii) muscle pressure does not establish instability with minimal interaction of tongue and lips on unaltered tooth position.^16^ Maintenance or minor change in intercanine width has also been reported.^17^ Existing systematic reviews on MIE were overall reviews^21-23^ or comparisons have been made with interproximal reduction.^24^ This systematic review assessed the stability of treatment results analyzed by considering parameters like intercanine width and peer assessment rating (PAR) scores after MIE in orthodontic patients.

## Methods

###  Protocol and registration

 The Preferred Reporting Items for Systematic Reviews and Meta-analyses (PRISMA) protocolwas followed, and the systematic review was registered with PROSPERO (CRD42020196379).

###  Eligibility criteria

 Studies fulfilling the following criteria were included:

1. Participants: Orthodontic patients evaluated during the retention period
2. Interventions: Orthodontic treatment with MIE
3. Comparisons: Patients treated orthodontically with all four premolar extractions (PE) or non-extraction (NE)
4. Primary Outcome: Changes in intercanine width
5. Secondary outcome: PAR index scores
6. Study design: Longitudinal studies, either prospective or retrospective

The exclusion criteria were studies without a control group, case reports, case series with no statistical analysis, reviews, expert opinions, and letters to the editor.

###  Data sources

 PubMed, Cochrane Library, Science Direct, Google Scholar, Ovid, and SciELO were systematically searched without restrictions in the year of publication or language up to August 2022.

###  Search strategy 

 The search strategy followed for the six search engines is presented in Table 1.

**Table 1:** Search strategy followed in different databases

**Search engines**	
PubMed	(incisor extraction[Title/Abstract]) AND (intercanine width[Title/Abstract] OR stability[Title/Abstract] OR PAR index[Title/Abstract] OR irregularity[Title/Abstract] OR relapse[Title/Abstract] OR outcome*[Title/Abstract] OR Peer Assessment Rating[Title/Abstract])
Cochrane	(mandibular incisor extraction) AND (intercanine width) OR (post-orthodontic stability)
Google Scholar	"Mandibular incisor extraction" and changes in intercanine width or post-treatment stability in orthodontic patients
SciELO	(ab:(incisor extraction AND intercanine width)) OR (ab:(incisor extraction AND stability)) OR (ab:(incisor extraction AND PAR index)) OR (ab:(incisor extraction AND irregularity)) OR (ab:(incisor extraction AND relapse)) OR (ab:(incisor extraction AND outcome)) OR (ab:(incisor extraction AND outcomes)) OR (ab:(incisor extraction AND Peer Assessment Rating))
Science Direct	Mandibular incisor extraction, changes in intercanine width, or post-orthodontic stability
Ovid	((intercanine width or stability or irregularity or relapse or outcome or Peer Assessment Rating) and incisor extraction)
Web of Science	ALL = (“incisor extraction”) AND ALL = (intercanine width OR stability OR PAR index OR irregularity OR relapse OR outcome OR outcomes OR Peer Assessment Rating)

###  Study selection 

 The titles and abstract results were screened, and irrelevant articles and duplicates were excluded. Three reviewers independently assessed the articles for eligibility and obtained full texts. References were hand-searched for additional relevant studies. Finally, articles that met the above inclusion criteria were selected.

###  Data collection and data items

 Three reviewers independently collected data using a data collection form with a standardized table. All linear measurements and percentage scores measured before treatment, after treatment, and after the retention period were extracted. The data were compared for accuracy, and any conflicts were resolved by reexamining the original study and discussions between the reviewers until a consensus was achieved.

###  Assessment of bias risk within studies

 The risk of bias in nonrandomized studies was assessed using a modified version of the Newcastle-Ottawa Scale (NOS).^25^ The reviewers used a star system and assessed eight domains and three criteria, patient selection (maximum of four points), comparability (two points), and assessment of outcome (three points for exposure/outcome), and then the overall bias was judged. Four stars or less indicated low quality or high ROB, 5‒6 stars indicated moderate ROB, and > 7 indicated low ROB.

###  Evaluation of the level of evidence

 The level of evidence was assessed using Grading of Recommendations, Assessment, Development, and Evaluation (GRADE) (https://gradepro.org/).^26^ For each outcome examined, the GRADE assesses the number of studies included, the studies’ designs, risk of bias, inconsistency, indirectness, imprecision, and other considerations (such as publication bias). Depending on the seriousness of the limitation in each one of these domains, the evidence could be downgraded by 1 or 2 levels. Based on this assessment, the certainty of evaluating the outcome could be very low, low, moderate, or high quality.

###  Summary measures

 The outcome measures were differences in means of the intercanine width (mm) and the percentages of mean reduction in PAR scores. Meta-analysis was performed using Review Manager (RevMan) version 5.4 (The Nordic Cochrane Centre, Copenhagen, Denmark).

###  Synthesis of results

 The data extracted were descriptively tabulated. Meta-analysis results were graphically represented with forest plots. Clinical heterogeneity was assessed by comparing the study design, control groups, and methodologies. Statistical heterogeneity was evaluated with the Cochrane Tau^2^, χ^2^ and I^2^ statistics (low = 25%, moderate = 50%, and high = 75%). Studies were statistically evaluated, and significance was established at *P* < 0.05. I^2^ with a 95% confidence interval (CI) and the *P *value for χ^2^ were interpreted together. Random effects meta-analysis was carried out when there were high levels of clinical or statistical heterogeneity.

## Results

###  Study selection

**Figure 1:**
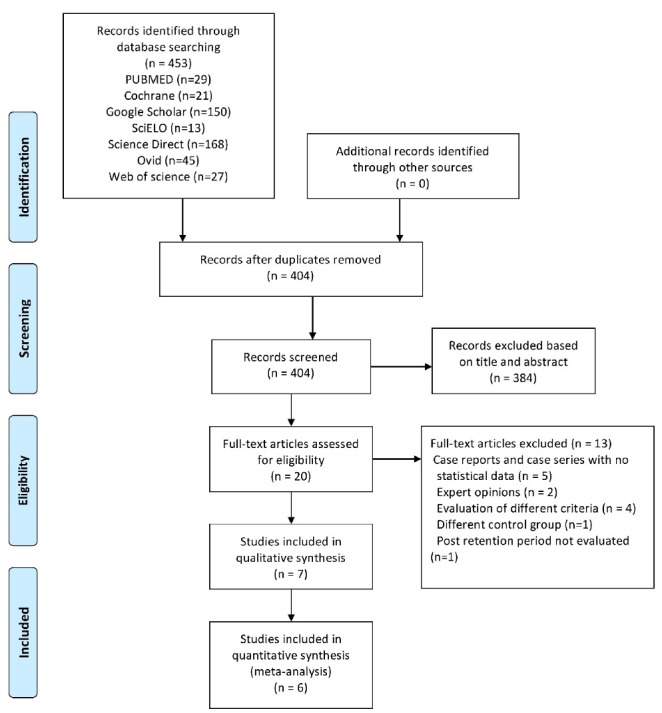
PRISMA flowchart of article retrieval.

 The search yielded no results of randomized control trials or prospective studies; all the included studies were nonrandomized retrospective studies. The search strategy yielded 453 studies from electronic databases and hand searches. Removing duplicates and applying eligibility criteria resulted in 20 articles for full-text evaluation. Seven articles were included in this systematic review and subjected to data collection and quantitative synthesis.^18-32^ The complete search strategy and reasons for exclusion after full-text assessment are provided in the PRISMA flowchart (Figure 1).

###  Study characteristics

Table 2provides descriptive characteristics of the studies included. Eighty-four individuals had undergone single incisor extraction in three studies, with intercanine width as one of the parameters evaluated^18,27,28^ and 100 individuals in the studies evaluating the PAR index.^29-32^ The participants were adults with an age range of 18‒35 years, treated with fixed appliances. Malocclusion involved was predominantly class I, and two studies evaluated class II and class I malocclusion.^18,29^ Post-retention follow-up varied vastly from 3 to 12 years among studies assessing intercanine width.^18,27,28^ Riedel et al^18^ used a removable retainer worn for two years,and Mahmoudzadeh et al^27^ used a clear or Hawley’s retainer. The patients were evaluated after 3.5 years,and all the patients had lingually bonded retainers for one year in the study by Verma and Jain et al.^28^ PAR scores were evaluated before and after treatment with no post-retention follow-up and no information on retention protocol.^29-32^

**Table 2:** Summary of the study characteristics of the included studies

**Studies**	**Study design**	**Participants**	**Intervention**	**Control**	**Outcome**
Riedel et al, 1992^18^	Retrospective study	107 Patients	42 patients 24 patients – single mandibular incisor extraction group 18 patients – two mandibular incisor extraction group (15 males, 27 females) mean age: 35.3 (24.10-57.8) years	65 previously reported PE cases (24 males, 41 females) mean age 30.1 (25-43.4) years	Intercanine width decreased during treatment and continued to decrease post-retention in most cases. (statistically significant) More stable than PE cases.
Mahmoudzadeh et al, 2018^27^	Retrospective study	120 Patients	40 patients in MIE group (31 females, 9 males) mean age: 21.6 ± 4 years	NE group: 40 patients (33 females, 7 males) mean age: 24 ± 6 years	Intercanine width decreased during treatment and continued to decrease post-retention in the MIE group. There was not much difference in the intercanine width in the NE and PE groups. There was no significant difference among the means in the three study groups.
				PE group: 40 patients (35 females, 5 males) mean age: 22.9 ± 5 years	
Verma and Jain, 2022^28^	Retrospective study	32 Patients	Lower incisor extraction protocol (n= 17)	NE protocol (n= 15)	Intercanine width increased significantly in the NE group post-treatment, and there was a significant relapse during post-retention follow-up. Overall the intercanine width reduced or remained unchanged in the incisor extraction group.
Ileri et al, 2012^29^	Retrospective study	60 Subjects	Extraction of a lower incisor group (MIE) 20 patients (13 females and 7 males)	Extraction of four first premolars group (PE) 20 patients (13 females and 7 males)	The percentage PAR reduction was lesser than in other groups. Orthodontic treatment without extraction had a better treatment outcome than the four-first PE and single lower incisor extraction protocols in Class I cases with moderate to severe mandibular anterior crowding.
				NE group 20 patients (13 females and 7 males)	
Kamal et al, 2017^30^	Retrospective study	108 Patients	MIE group 36 patients Age: 19.0 ± 2.3 years.	NE group, 36 patients Age: 18.9 ± 4.1 years	Differences between NE and MIE groups, and PE and MIE groups were statistically significant. Difference between NE and PE (not statistically significant) Percentages of improvement in PAR scores showed no significant difference among patients treated with NE, PE, and MIE. Mean improvements in the maxillary and mandibular anterior segment were greater in the MIE group.
				PE group, 36 patients Age: 19.2 ± 3.6 years	
Lee et al, 2019^31^	Retrospective study	28 Patients	MIE cases (n = 14)	NE controls (n = 14)	There were no significant differences in the treatment outcomes of orthodontic cases treated with MIE or NE.
Maaz et al, 2022^32^	Cross-sectional study	90 Patients	MIE cases (n = 30) Mean age: 20.21 ± 3.00 years	NE cases (n = 30) Mean age: 20.52 ± 4.08 years	There were significant differences in the percentage improvement for the PAR (*P* = 0.010) NE, PE, and MIE showing median percentage improvements of 82.1%, 91.3%, and 74.1%, respectively.
				PE cases (n = 30) Mean age: 20.42 ± 3.46 years	

PAR, peer assessment rating; PE, premolar extraction; MIE, mandibular incisor extraction; NE, non-extraction.

###  Risk of bias within studies 

Table 3 presents the risk of bias for each study. Riedel et al^18^ compared intercanine width in the post-retention period of patients who underwent lower incisor extraction. Data from previous research showed that this lack of a control group from a similar population and time led to a high risk of bias. The overall assessment showed a low risk of bias in four of the seven studies,^28,30-32^ and two studies had a moderate risk of bias.^27,29^

**Table 3:** Newcastle Ottawa Scale for observational studies

**Studies**	**Selection** **(maximum 4 stars)**				**Comparability** **(maximum 2 stars)**	**Outcome (maximum 3 stars)**			**Total stars (9)**	**Risk of bias**
Retrospective studies	Representativeness of the exposed cohorts	Selection of the non-exposed cohort	Ascertainment of exposure	Justification of study sample size	Comparability of cohorts on the basis of design or analysis	Assessment of outcome	Was follow-up long enough for the outcome to occur	Adequacy of follow-up of cohorts		
	a) Truly representative of the average in the target population* (all subjects or random sampling); b) Somewhat representative of the average in the target population* (nonrandom sampling); c) Selected group of patients; d) No description of the sampling strategy.	a) drawn from the same community as the target population*; b) drawn from a different source. c) no description of the derivation of the comparison population.	a) Secure records (e.g., surgical records)* b) Structured interview* c) Written self-report d) no description	a) Yes* b) No	The subjects in different outcome groups are comparable based on the study design or analysis. Confounding factors are controlled. a) The study controls for age and type of malocclusion*; b) The study controls for retention.* c) Study does not control for any of the above	a) Independent blind assessment*; b) Record linkage*; c) Self-report; d) No description.	a) Yes-after completion of orthodontic treatment* b) No	a) Complete follow-up - all subjects accounted for* b) Subjects lost to follow-up unlikely to introduce bias - small number lost (follow up, or description provided of those lost)* c) Follow-up rate and no description of those lost d) No statement		
Riedel et al, 1992^18^	c	b	a*	b	c	b*	a*	b*	4	High
Mahmoudzadehet al, 2018^27^	c	a*	b*	a*	b*	b*	a*	c	6	Moderate
Verma and Jain, 2022^28^	c	a*	a*	a*	a*,b*	b*	a*	b*	8	Low
Cross sectional studies	Representativeness of the exposed cohorts	Selection of the non-exposed cohort	Ascertainment of exposure	Justification of study sample size	Comparability of cohorts on basis of design or analysis	Assessment of outcome	Same method of ascertainment for exposed and non-exposed group	Statistical test		
	a) Truly representative of the average in the target population* (all subjects or random sampling); b) Somewhat representative of the average in the target population* (nonrandom sampling); c) Selected group of patients; d) No description of the sampling strategy.	a) Drawn from the same community as the target population*; b) Drawn from a different source. c) No description of the derivation of the comparison population.	a) secure record (e.g. surgical records) * b) structured interview * c) written self-report d) no description	a) Justified and satisfactory. * b) Not justified.	The subjects in different outcome groups are comparable, based on the study design or analysis. Confounding factors are controlled. a) The study controls for age, sex*; b) Type of malocclusion.* c) Study does not control for any of the above	a) Independent blind assessment*; b) Record linkage*; c) Self-report; d) No description.	a) Yes* b) No	a) The statistical test used to analyze the data is clearly described and appropriate, and the measurement of the association is presented, including confidence intervals and the probability level (p value).* b) The statistical test is not appropriate, not described or incomplete.		
Ileri et al, 2012^29^	c	a*	a*	b	a*	b*	a*	a*	6	Moderate
Kamal et al,2017^30^	c	a*	a*	a*	a*,b*	b*	a*	a*	8	Low
Lee et al, 2019^31^	c	a*	a*	a*	a*,b*	b*	a*	a*	8	Low
Maaz et al 2022^32^	c	a*	a*	a*	a*,b*	b*	a*	a*	8	Low

###  Synthesis of results

####  Results of individual studies and meta-analysis

Table 4 summarizes the results of variables assessed in the studies.

**Table 4:** Results of the individual studies

**Changes in intercanine width**					
**Studies**	**Malocclusion treated**	**Treatment duration**	**Intercanine width**	**Difference**	**Significance of difference** ***P*** ** value**
Riedel et al, 1992^18^	Class I Class II div 1 Class II div 2	Post-retention period: 12.9 years (6.6-24)	Single incisor extraction cases: Pre-treatment: 24.37 ± 2.53 mm Post-treatment: 22.77 ± 1.10 mm Post-retention: 21.64 ± 1.41 mm	D1 -1.63 ± 2.22 mm D2 -1.13 ± 0.95 mm	*P*0.05* D1 and D2
			PE1: scatter diagram of intercanine width	D2 -2.02 ± 1.57 mm	*P* < 0.0001***
Mahmoudzadeh et al, 2018^27^	Class I or Class II Division 1 malocclusion	Retention period: 8 months ± 4.7 Post-retention period: 3.35 ± 1.48 years	MIE cases: Pre-treatment: 25.56 ± 2.19 mm Post-treatment: 23.02 ± 2.02 mm Post-retention: 22.37 ± 1.24 mm	D2 -0.65 ± 1.58 mm	*P* = 0.012 *
			NE cases: Pre-treatment: 26.30 ± 1.58 mm Post-treatment: 26.61 ± 1.79 mm Post-retention: 25.94 ± 1.61 mm	D2 -0.67 ± 1.18 mm	*P* = 0.001*
			PE cases: Pre-treatment: 26.82 ± 2.51 mm Post-treatment: 26.80 ± 1.56 mm Post-retention: 26.27 ± 1.85 mm	D2 -0.53 ± 1.14 mm	*P* = 0.006*
					Between groups *P* = 0.870 NS
Verma and Jain, 2022^28^	Class I malocclusion	Post-retention period: 1 year after completion of treatment	MIE cases: Pre-treatment: 23.5 ± 1.7 mm	D1 -0.94 ± 0.35 mm D2 -0.01 ± 0.05 mm D3 -0.95 ± 0.47 mm	P < 0.05 *
			NE cases: Pre-treatment: 22.8 ± 2.9 mm	D1 2.01 ± 0.18 mm D2 -1.37 ± 0.40 mm D3 0.65 ± 0.58 mm	
**Changes in the PAR index scores**					
**Studies**	**Malocclusion treated**	**Treatment duration**	**PAR Scores**	**PAR improvement 1percentage**	**Significance of difference** ***P*** ** value**
Ileri et al, 2012^29^	Class I malocclusion and moderate crowding	1.6 ± 0.9 years	MIE cases: Pre-treatment: 21.5 ± 1.5 Post-treatment: 3.8 ± 3.52	80.3 ± 18%	*P* < 0.05* Between groups MIE & NE *P* = 0.047*, others NS
		2 ± 0.4 years	PE cases: Pre-treatment: 27 ± 6.2 Post-treatment: 3.5 ± 3.19	87.7 ± 10.2%	
		1.3 ± 0.4 years	NE cases: Pre-treatment: 17.1 ± 5.7 Post-treatment: 1.4 ± 1.14	91.2 ± 9.2%	
Kamal et al, 2017^30^	Class I malocclusion		MIE cases: Pre-treatment: 33.3 ± 10.4 Post-treatment: 9.1 ± 7.5	70.6 ± 24.1%	*P* < 0.001** Between groups *P* = 0.351 NS
			PE cases: Pre-treatment: 23.5 ± 9.4 Post-treatment: 5.5 ± 3.7	73.1 ± 19.4%	
			NE cases: Pre-treatment: 20.5 ± 9.5 Post-treatment: 4.7 ± 4.2	75.8 ± 25.8%	
Lee et al, 2019^31^	Class I malocclusion or a mild tendency toward Class III, moderate crowding (4–8 mm) in the lower arch		MIE cases: Pre-treatment: 18.8 ± 7.3 Post-treatment: 3.8 ± 3.1 Changes: 15.0 ± 8.6	73.2 ± 32.1%	Between groups *P* = 0.874 NS
			NE cases: Pre-treatment: 16.7 ± 8.4 Post-treatment: 4.0 ± 4.4 Changes: 12.7 ± 8.0	75.6 ± 25.2%	
Maaz et al 2022^32^	Class I malocclusion		MIE cases:	80%	*P* = 0.010 * Between groups MIE & PE *P* = 0.002*, others NS
			PE cases:	98%	
			NE cases:	89.1%	

PAR, peer assessment rating; PE, premolar extraction; MIE, mandibular incisor extraction; NE, non-extraction. D1: difference from post-treatment to pre-treatment, D2: difference from post-retention to post-treatment, D3: difference from post-retention to pre-treatment. Statistically significant at * *P* < 0.05; **P < 0.001; ***P < 0.0001; NS, No significant.

 A reduction in intercanine width during treatment and post-retention was reported in the MIE group.^18,27,28^ Two studies showed a decrease in intercanine width post-treatment and post-retention.^18,27^ Among them, one reported intercanine width reduction post-retention in single incisor extraction (1.13 ± 0.95 mm) and two incisor extraction (1.39 ± 1.19 mm).^18^ Another study by Mahmoudzadeh et al^27^ reported a post-retention decrease in intercanine width in MIE (0.65 ± 1.5 mm), NE (0.67 ± 1.18 mm) and PE (0.53 ± 1.14 mm).In the study by Vermaet al,^28^ there was an increase in intercanine width in the NE group, and intercanine width decreased by 0.94 mm in MIE.

 When PAR scores were evaluated between pre-treatment and post-treatment periods, they were significantly reduced post-treatment, and percentage improvement was reported.^29-32^ The mean percentage improvement of PAR reported by Ileri et al^29^ in MIE was 80.3%, NE was 91.2%, and PE was 87.7%.Kamal et al^30^ reported percentage improvements of 70.6 ± 24.1% in MIE, 75.8 ± 25.8% in NE and 73.1 ± 19.4% in PE.Lee et al^31^ reported an improvement of 73% in MIE and 76% in the NE group. Maaz et al^32^ reported an 80% PAR percentage improvement in MIE, 98% in PE, and 89.1% in NE.

**Figure 2:**
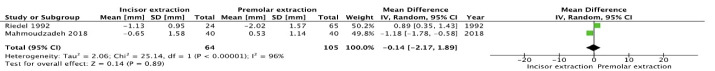
Forest plot comparing pre-treatment and post-retention intercanine (IC) width reduction in mandibular incisor extraction compared to premolar extraction.

**Figure 3:**
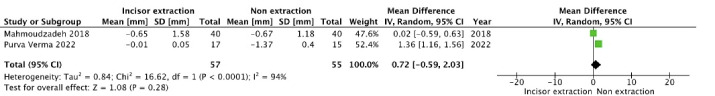
Forest plot comparing post-treatment and post-retention intercanine (IC) width reduction in mandibular incisor extraction compared to non-extraction.

 There was relative clinical homogeneity in the assessment methods and reporting the mean changes of the intercanine and PAR scores in the mandibular incisor and PE groups and the NE group in six studies included in the meta-analysis.^18,27-31^ Forest plot was used to compare the mean difference (MD) in intercanine width changes between the post-treatment and post-retention periods in incisor extraction with PE (Figure 2) and NE (Figure 3) to quantitatively determine whether there was a significant reduction post-retention. A statistically significant decrease in mean intercanine widthwas seen between the premolar and incisor extraction groups (MD = 0.14, 95% CI: -2.17–1.89; *P* < 0.00001). When the intercanine width changes of the incisor extraction and NE groups were compared, a statistically significant reduction in mean intercanine widthwas evident in the incisor extraction group compared to the NE group (MD = 0.72, 95% CI: -0.59–2.03; *P* < 0.00001). Both comparisons showed high heterogeneity, and a random effects model was used. Standard random-effects approaches added a common component of variance to each study weight in the presence of heterogeneity to account for the variation in treatment effects between trials, and the relative weights were more balanced than those awarded under fixed effects.^33^

**Figure 4:**
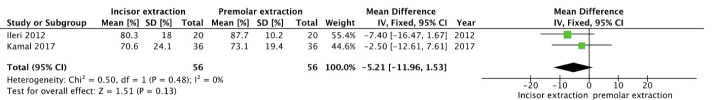
Forest plot comparing PAR reduction percentages of mandibular incisor extraction and premolar extraction.

**Figure 5:**
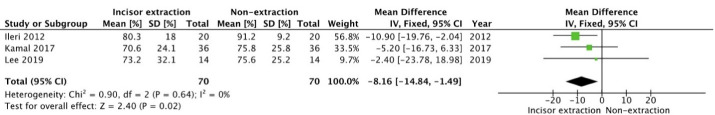
Forest plot comparing PAR reduction percentages of mandibular incisor extraction and non-extraction.

 For the clinical relevance of improvement in treatment results, PAR reduction percentages of MIE were compared individually with four PEs (Figure 4) and NE (Figure 5). The mean difference was calculated, and there was no significant differencein PAR reduction percentage between mandibular incisor and four PE groups (MD = -5.21, 95% CI: -11.96–1.53; *P* = 0.48) or between lower incisor extraction and NE groups (MD = -8.16, 95% CI: -14.84 – -1.49; *P* = 0.64). The forest plots showed low heterogeneity, with the left side favoring lower incisor extraction and the right side favoring the premolar or NE group.

###  Quality analysis

Table 5 summarizes the quality assessment of outcomes from included articles using GRADE. The overall effect was considered very low certainty for studies evaluating the intercanine width and low for the PAR index. In general, observational studies showed inconsistency in retention methods and follow-up durations. Riedel et al did not have a uniform control group, and they were the main factors for the limited quality of evidence.

**Table 5:** GRADE assessment

**Certainty assessment**							**№ of patients**		**Effect**		**Certainty**	**Importance**
**No. of studies**	**Study design**	**Risk of bias**	**Inconsistency**	**Indirectness**	**Imprecision**	**Other considerations**	**Incisor extraction**	**PE or NE**	**Relative** **(95% CI)**	**Absolute** **(95% CI)**		
**Intercanine Width (follow-up: range 1 to 10 years)**												
3	observational studies	Not serious	Not serious	Serious^a^	Not serious	None	81	160	-	Not estimable	⨁◯◯◯ Very low	
**PAR Index**												
4	observational studies	Not serious	Not serious	Not serious	Not serious	None	100	186	-	Not estimable	⨁⨁◯◯ Low	

CI, confidence interval; PAR, peer assessment rating; PE, premolar extraction; NE, non-extraction. Question:Incisor extraction compared to PE or NE in treatment results and stability. ^a^Riedel study showed serious indirectness in reporting data, and the sample size is heterogeneous.

###  GRADE Working Group grades of evidence

*High certainty:* We are very confident that the true effect lies close to that of the estimate of the effect.

*Moderate certainty:* We are moderately confident in the effect estimate: The true effect is likely to be close to the estimate of the effect, but there is a possibility that it is substantially different.

*Low certainty:* Our confidence in the effect estimate is limited: The true effect may be substantially different from the estimate of the effect.

*Very low certainty:* We have very little confidence in the effect estimate: The true effect is likely to be substantially different from the estimate of effect.

## Discussion

 This systematic review assessed the treatment outcomes and stability in studies where MIE was adopted as a treatment option. On screening the available literature, seven studies met the inclusion criteria of this systematic review. All seven studies were retrospective observational studies and were of very low to low evidence, which was assessed by the GRADE tool. Mandibular intercanine width was one of the outcomes assessed in studies in which MIE was carried out.^18,27,28^ The stability of treatment was assessed as maintaining dental arch form and arch widthis essential for stable orthodontic treatment results.^34^ Some studies reported pre-treatment and post-treatment changes in PAR scores as one of the most common tools used in orthodontics to validate treatment outcomes.^29-32^

 Maintaining the intercanine width or minimal alterations post-retention can be considered one key factor for stable results. On observation of treatment changes and post-retention changes, a predominant reduction was found to occur between pre-treatment and post-treatment in MIE(Table 4).This can be attributed to the fact that during treatment with incisor extraction, intercanine width tends to decrease as tooth material reduction occurs upon the removal of the mandibular incisor. Dacre^11^ studied the untreated cases compared to incisor extraction, and a reduction in intercanine width of 0.3 ± 1.30 mm was observed in the long term, even in the absence of treatment, suggesting a decrease in intercanine width with age.According to Burke et al,^35^ mandibular intercanine width tends to expand during treatment on the order of 1‒2 mm and contract post-retention to approximately the original dimension, regardless of patient diagnostic and treatment modalities.

 Riedelet al^18^ reported a reduction in both single and two MIE groups and found it significantly less than PE cases with no significant correlation between treatment and post-retention changes. They suggested that it might satisfy the requirements of maintaining arch form without expanding intercanine width, while in PE therapy, an increase in intercanine width might be required to gain alignment. Post-treatment and post-retention evaluations of intercanine width showed some reduction in the evaluated studies.^18,27,28^ In the meta-analysis, there was a significantly greater reduction in intercanine width in the PE group compared to MIE (Figure 2). Mahmoudzadeh et al^27^ reported no statistically significant difference in the intercanine width in post-retention changes between incisor extraction, NE, and PE.

 According to Vermaet al,^28^ there was a mild decrease in intercanine width in the incisor extraction group, which was maintained post-retention, whereas, in the NE group, there was an increase in intercanine width that reduced to a greater extent post-retention. When this increase in intercanine distance was analyzed in NE cases, it was noticed that the NE control group was treated by self-ligating brackets, which might be a reason for this increase. Interproximal reduction is sometimes carried out in NE treatment to address Bolton’s discrepancies. A systematic review showed that both interproximal wear and incisor extraction are effective in treating moderate anterior crowding, and one of the studies reviewed reported that the stability of treatment outcomes was evident as there was no change in intercanine width in patients who underwent proximal stripping.^24^ Similarly, in the present research, a meta-analysis comparing the incisor extraction and NE groups showed a greater reduction in intercanine width in the incisor extraction group(Figure 3).When MIE was compared to PE and NE treatment protocol (Figures 2 and 3), post-retention changes in intercanine width in MIE cases were significantly greater than in NE and less than in PE groups. Extraction of mandibular incisors in selected cases of Bolton excess in the mandibular anterior region to gain space to alleviate crowding is thus justified.

 Ileri et al^29^ reported that the mean improvement in PAR scores was the highest in NE followed by PE and least in the MIE group, and the same results were reported by Maaz et al.^32^ The least reduction in MIE was thought to be probably due to increasing overjet and overbite. The meta-analysis comparing PAR score improvement percentages of MIE and NE, MIE, and PE showed no significant differences(Figures 4 and 5). These findings were confirmed by Kamal et al^30^ and Lee et al.^31^

 All the studies assessing PAR scores^29^ showed > 70% reduction in PAR percentage scores with MIE, which is considered a high standard outcome according to Richmond et al.^36^ None of the studies assessed PAR post-retention but the PAR index, in general, can be considered an indicator of stability as PAR index follow-up studies have shown good stability up to 76.3%.^37^

 The positive outcomes observed through this systematic review suggest that MIE can be a clinically effective treatment modality in carefully selected cases. Orthodontists should pursue MIE as a valuable option to provide results in cases like class I malocclusion with mild to moderate crowding, mild class III tendency, acceptable soft tissue profile, moderate overjet and overbite, and Bolton’s discrepancies. Negligible relapse in the anterior area, maintenance of soft tissue profile, and no compromise in esthetics or function can be worthwhile results achieved with shorter treatment time and simpler mechanics.

## Limitations and Future Directions

 All the studies included in this systematic review were retrospective. The paucity of randomized controlled trials proved to be a limitation.

 The PAR index is not an optimal tool for evaluating treatment benefits and does not consider all factors important for the total quality of treatment.^38^ PAR provides only a general impression of dental arches, and no individual dental variables are considered.

 As there is a scarcity of studies on the assessment of post-treatment stability, and study designs also greatly vary concerning post-retention stability of intercanine width, drawing a conclusion remains challenging, indicating the need for more studies, controlled trials with matched controls, and similar pre-treatment characteristics and post-retention period. Maintaining records and longer post-retention follow-ups are thus emphasized to provide better long-term studies and evidence.

## Conclusion

 The retrospective studies in this systematic review provided limited quality evidence, making it difficult to draw significant evidence-based conclusions. More well-designed studies are required for a definitive conclusion on long-term stability. Thus the conclusions derived were as follows:

1. There was a reduction in intercanine width during post-retention, significantly higher in PE than MIE. However, there was significantly less intercanine width reduction in the NE group than in incisor extraction.
2. The high standard of outcome analyzed with PAR scores suggested MIE as a valid treatment option, and the results were comparable with PE and NE groups, with no significant difference between the groups.
3. One treatment option cannot be better than the other, and treatment choices should be made according to the clinical situation.

## Competing Interests

 The authors declare that they have no competing interests with regard to the authorship and/or publication of this article.

## Ethical Approval

 Not applicable.

## Funding

 There were no sources of funding for the study.
